# Four Weeks of Detraining Induced by COVID-19 Reverse Cardiac Improvements from Eight Weeks of Fitness-Dance Training in Older Adults with Mild Cognitive Impairment

**DOI:** 10.3390/ijerph18115930

**Published:** 2021-05-31

**Authors:** Achraf Ammar, Omar Boukhris, Nicole Halfpaap, Berit Kristin Labott, Corinna Langhans, Fabian Herold, Bernhard Grässler, Patrick Müller, Khaled Trabelsi, Hamdi Chtourou, Piotr Zmijewski, Tarak Driss, Jordan M. Glenn, Notger G. Müller, Anita Hoekelmann

**Affiliations:** 1 Institute of Sport Science, Otto-von-Guericke University, 39106 Magdeburg, Germany; nicole.halfpaap@ovgu.de (N.H.); berit.labott@ovgu.de (B.K.L.); corinna.langhans@ovgu.de (C.L.); bernhard.graessler@ovgu.de (B.G.); anita.hoekelmann@ovgu.de (A.H.); 2 Interdisciplinary Laboratory in Neurosciences, Physiology and Psychology: Physical Activity, Health and Learning (LINP2), UFR STAPS, UPL, Paris Nanterre University, 92000 Nanterre, France; tarak.driss@parisnanterre.fr; 3 Physical Activity, Sport, and Health, UR18JS01, National Observatory of Sport, Tunis 1003, Tunisia; omarboukhris24@yahoo.com (O.B.); h_chtourou@yahoo.fr (H.C.); 4 High Institute of Sport and Physical Education of Sfax, University of Sfax, Sfax 3000, Tunisia; trabelsikhaled@gmail.com; 5 German Center for Neurodegenerative Diseases (DZNE), 39104 Magdeburg, Germany; Fabian.herold@dzne.de (F.H.); patrick.mueller@dzne.de (P.M.); notger.mueller@dzne.de (N.G.M.); 6 Department of Neurology, Medical Faculty, Otto von Guericke University, Leipziger Str. 44, 39120 Magdeburg, Germany; 7 Research Laboratory, Education, Motricity, Sport and Health, EM2S, LR19JS01, University of Sfax, Sfax 3000, Tunisia; 8 Jozef Pilsudski University of Physical Education in Warsaw, 00-809 Warsaw, Poland; piotr.zmijewski@insp.waw.pl; 9 Exercise Science Research Center, Department of Health, Human Performance and Recreation, University of Arkansas, Fayetteville, AR 72701, USA; jordan@neurotrack.com; 10 Neurotrack Technologies, 399 Bradford St, Redwood City, CA 94063, USA; 11 Center for Behavioral Brain Sciences (CBBS), Brenneckestraße 6, 39118 Magdeburg, Germany

**Keywords:** pandemics, training cessation, combined training, aerobic, strength, aging, physical activity, cardiovascular health, heart rate, HRV, performance, responsiveness

## Abstract

Physical training is considered as a low-cost intervention to generate cardioprotective benefits and to promote physical and mental health, while reducing the severity of acute respiratory infection symptoms in older adults. However, lockdown measures during COVID-19 have limited people’s opportunity to exercise regularly. The aim of this study was to investigate the effect of eight weeks of Fitness and Dance training, followed by four weeks of COVID-19-induced detraining, on cardiac adaptations and physical performance indicators in older adults with mild cognitive impairment (MCI). Twelve older adults (6 males and 6 females) with MCI (age, 73 ± 4.4 y; body mass, 75.3 ± 6.4 kg; height, 172 ± 8 cm; MMSE score: 24–27) participated in eight weeks of a combined Fitness-Dance training intervention (two sessions/week) followed by four weeks of training cessation induced by COVID-19 lockdowns. Wireless Polar Team Pro and Polar heart rate sensors (H10) were used to monitor covered distance, speed, heart rate (HR min, avg and max), time in HR zone 1 to 5, strenuousness (load score), beat-to-beat interval (max RR and avg RR) and heart rate variability (HRV-RMSSD). One-way ANOVA was used to analyze the data of the three test sessions (T1: first training session, T2: last training session of the eight-week training program, and T3: first training session after the four-week training cessation). Statistical analysis showed that eight weeks of combined Fitness-Dance training induced beneficial cardiac adaptations by decreasing HR (HR min, HR avg and HR max) with *p* < 0.001, ES = 0.5–0.6 and Δ = −7 to−9 bpm, and increasing HRV related responses (max and avg RR and RMSSD), with *p* < 0.01 and ES = 0.4. Consequently, participants spent more time in comfortable HR zones (e.g., *p* < 0.0005; ES = 0.7; Δ = 25% for HR zone 1) and showed reduced strenuousness (*p* = 0.02, Δ = −15% for load score), despite the higher covered total distance and average speed (*p* < 0.01; ES = 0.4). However, these changes were reversed after only four weeks of COVID-19 induced detraining, with values of all parameters returning to their baseline levels. In conclusion, eight weeks of combined Fitness-Dance training seems to be an efficient strategy to promote cardioprotective benefits in older adults with MCI. Importantly, to maintain these health benefits, training has to be continued and detraining periods should be reduced. During a pandemic, home-based exercise programs may provide an effective and efficient alternative of physical training.

## 1. Introduction

In the last century, life expectancy has increased worldwide (~27 years), with an increasing number of adults aged over 60 years old [1]. In 2018, nearly one fifth (19%) of the European population was aged ≥65 [2]. This number is expected to reach 25.6% by 2030 and 29.5% by 2060 [3]; as a result, our societies need to deal with the simultaneous rise in age-related chronic diseases (i.e., neurodegenerative diseases) [4,5,6]. Neurodegenerative diseases have tremendous physical, psychological, social, and economical impacts, with previous reports identifying severe age-related cognitive decline (ARCD) as a primary risk factor [5].

The etiology of age-associated cognitive decline is complex and multifactorial [7]; however, primary risk factors associated with age-associated cognitive declines include altered cardiovascular parameters and associated disease states (e.g., hypertension, obesity, diabetes, etc.) [8,9]. These risk factors may promote atherosclerosis and microvascular diseases [10], contributing to neuronal degeneration and hypoperfusion [11]. Another risk factor related to ARCD is physical inactivity [12]. A sedentary lifestyle is a primary contributor to increased rates of overweight and obesity among older adults [13], who represent a population at higher risk for cognitive decline due to the adverse effects of excess body weight on, for instance, oxidative stress [14], inflammation [15], and vascular function [16].

Conversely, physical training is considered as a low-cost intervention for the prevention and management of cardiac diseases [17] and has been suggested to convey a preventive effect against ARCD [18]. In this context, 10 weeks of moderate or high-intensity treadmill walking significantly improved aerobic capacity, represented by peak oxygen uptake, in people with cardiac co-morbidities [19]. Similarly, a physical training program was effective for (i) improving physical capacities such as endurance, balance, coordination and flexibility at six months in disabled older female cardiac patients [20], and (ii) reducing the odds of re-admission to hospital at 90 days in older patients hospitalized for heart failure [21]. With regard to cognitive function, available studies revealed that in older adults with mild cognitive impairments (MCI), physical training at a moderate intensity can improve performance in various cognitive domains (memory, executive functioning, attentional capacities, and processing speed) [22,23,24,25]. In this context, a large longitudinal study conducted by our research group showed increases in hippocampal volume and plasma brain-derived neurotrophic factor (BDNF) levels, and improvement in verbal memory and attentional performances following six months of Dance- or Aerobic-based exercises in healthy older adults [26,27,28,29,30]. Importantly, balance [29] and certain gait parameters (e.g., fall-risk related markers such as minimum foot-floor distance and non-linear gait stability) [31,32] were only improved following the Dance training program. These findings suggest that regular dancing training could contribute to the prevention of falls and of dementia (by stimulating neuroplasticity in certain brain regions [29]), thus fostering successful and autonomous aging. Investigating both cardiac-related output and cognitive parameters in healthy older adults, a more recent study showed that eight-weeks of concurrent training (aerobic and strength exercises) may improve aerobic capacity, operationalized by increasing maximal oxygen consumption (VO_2_max) and vigilance (related to executive functions) [33].

While the long-term cardiac and cognitive-promoting effects of physical exercise are encouraging in healthy and/or older adults with cardiovascular problems, the potential of regular physical training to counteract age-related cardiovascular and cognitive declines needs to be further confirmed in older adults who already exhibit cognitive decline such as MCI. To overcome this gap and taking into consideration that physical training has been suggested to promote physical health [34,35,36] and mental wellbeing [37,38] during the COVID-19 outbreak [39], our research group launched the “Dance against dementia (DiADEM)” RCT following the first wave of COVID-19 in Germany to investigate whether six-month combined Fitness and Dance training can improve cardiorespiratory, motor, and cognitive abilities in older-adults with MCI. Given that MCI is considered as a major risk factor for developing dementia, it was hypothesized that such an intervention in MCI patients may have the potential to prevent/delay the conversion from MCI to dementia.

Unfortunately, eight weeks after the start of the training intervention, in order to contain the second wave and curb the spread of COVID-19, public health authorities in Germany again imposed severe restrictions on public life, with stricter social-distancing policies implemented. In parallel with the national holidays (Christmas), these new restrictions induced four weeks of detraining. Following this detraining period, an updated hygienic concept (e.g., multiple training groups) was implemented due to stricter COVID-19 restrictions. With this unexpected situation, we sought to determine the consequences of training cessation in terms of cardiovascular adaptation. Available scientific literature reported a reversal of cardiovascular autonomic adaptations (e.g., VO2max, heart rate variability (HRV)) following four to eight weeks of detraining in healthy military sailors [40] or sedentary adults [41]. To our knowledge there is a lack of data investigating the retainability of cardioprotective benefits after training cessation in older adults with MCI.

Therefore, this study aims to investigate the effect of eight weeks of Fitness-Dance training, followed by four weeks of COVID-19 induced detraining, on cardiac adaptation and physical performance indicators in older adults with MCI.

## 2. Materials and Methods

The present study reports preliminary findings from the DiADEM RCT which began in October 2020 at the Department of Sport and Technology at the Otto von Guericke University Magdeburg in collaboration with the German Center for Neurodegenerative Diseases, Magdeburg, Germany, in accordance with the CONSORT statement [42] for RCT studies. The study protocol was approved by the Ethics Committee of the Otto von Guericke University (ref. n.: 17/20) and all procedures were performed in compliance with the latest version of the Declaration of Helsinki for conducting human experimentation. The study protocol was registered in the German Clinical Trials Register (DRKS-ID: DRKS00022575) on 5 August 2020 and was added to the Cochrane Central Register of Controlled Trials on 30 November 2020 (CENTRAL-ID: CN-02186572). Each participant was informed about the purpose, procedure, potential risks and benefits of the study and consequently signed an informed consent form before data collection.

**Figure 1:**
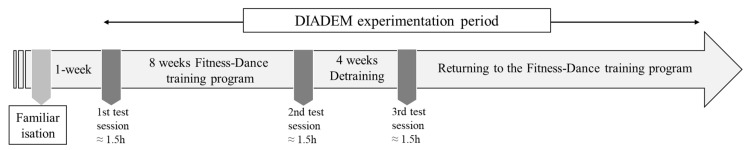
Study design.

Particularly, the present study focuses on participants’ physical performance and heart rate (HR) data collected during three test sessions separated by either an 8 week Fitness-Dance training program (between the 1st and 2nd test sessions) or a 4 week detraining induced by lockdowns & the national holidays (between the 2nd and 3rd test sessions) (Figure 1). These three test sessions are part of the 6 month DiADEM training program.

### 2.1. Participants’ Screening

Older adults with MCI were recruited through advertisements with flyers, posters and newspapers and using existing patient databases. Potential participants were screened for eligibility using the following inclusion criteria: Aged between 50 and 80 years old, and diagnosed with MCI. At first, participants had to report subjective memory complaints, then participants with pathological CERAD-plus results (scored 1.5 z-scores below the age- and education-adjusted reference sample in at least one subtest) were seen by an experienced neurologist who then decided whether participants fulfilled the criteria of MCI [43]. Additionally, to ensure that participants were primed physically to perform the training intervention, the Modified German version of Physical Activity Readiness Questionnaire (PAR-Q) was filled out before the start of the study [44]. Exclusion criteria were: other neurological diseases (e.g., epilepsy, multiple sclerosis), severe cardiac disease (e.g., severe heart failure, pacemaker, heart valve defect), psychiatric illnesses (e.g., schizophrenia, diagnosed depression with a score above 5 in the Geriatric Depression Scale (GDS)) [45], orthopedic diseases (e.g., broken bones in the last 6 months, symptomatic herniated disc), muscular disorders (e.g., myositis or tendovaginitis), severe endocrinological diseases (e.g., manifest thyroid dysfunction, obesity (BMI > 30), insulin-dependent diabetes mellitus type II), acute injuries or surgery (<6 months), consumption of illegal intoxicants or alcohol abuse, uncorrected visual or hearing impairment, color blindness/red-green weakness, and pregnancy or breastfeeding. Eligible participants were randomized to either an intervention group (to perform the Fitness-Dance training program), or to a control group (advised to follow their habitual daily routines).

### 2.2. The Fitness and Dance Training Programs

The intervention group received twice weekly 90 min training sessions (Wednesday and Friday). From 23 October (1st test session) to 18 December 2020 (2nd test session), the participants trained in one group together, following a hygienic concept due to the COVID-19 pandemic. After that, there was a break of 4 weeks due to a lockdown in Germany and the national holidays (Christmas). The 3rd test session was performed on 15 January 2021. Each session consisted of 10 min of warm-up with mobilization exercises, followed by coordinative exercises (35 min), including the learning of short Dance steps with constantly changing choreographies. These learning situations focused on elementary longitudinal turns, head-spins, shifts of center of gravity (COG), single-leg stances, skips and hops, different steps like chassée, mambo, cha cha, grapevine, and jazz square, to challenge the balance system and to stimulate participants’ memories. Afterwards, fitness Dance elements combining strength and aerobic exercises with Dance choreography (among others, step aerobics, AROHA^®^, Drums Alive^®^) were performed during 35 min. The training session ended with 10 min of whole-body stretching. Purposefully selected music was applied in all training sessions for motivational purposes. All participants wore the Polar H10 HR sensor (Polar, Kempele, Finland) during the training sessions. Participants’ heart rates, distance and speed were tracked with the Polar Team System. Rating of Perceived Exertion was collected immediately after each test session. The three test sessions were part of the Fitness-Dance training program. To avoid time of day effect (TOD) [46,47,48], all training/test sessions were performed in morning hours. For all test sessions, participants were asked to maintain normal sleep patterns [49] and not to ingest caffeinated food at least 4 h before their passage [50].

### 2.3. Measurements

#### 2.3.1. The Neuropsychological Test Battery “CERAD-Plus”

The German-language version of the CERAD-Plus (Consortium to Establish a Registry for Alzheimer’s Disease; developed by the Memory Clinic of the University Hospital Basel, Switzerland) is used as the standard procedure for clarifying and objectifying cognitive impairments in patients over 50 years.

The CERAD-Plus consists of the following tests: (i) CERAD test battery including Verbal fluency (Animals); Boston Naming Test; Mini–Mental State Examination (MMSE); Word list Learning; Word list recall, Word list intrusions; Word list savings; Word list recognizing; Figures drawing; Figures recall; Figures saving; [51] (ii) tests including Trail Making Test A and B and Phonematic fluid (S Words) test [52]. The areas of visio-constructive and spatial skills, linguistic and visual memory, word finding, language, fluency, attention/concentration, psychomotor speed, as well as executive functions such as organization, planning and flexibility are measured. The individual results are set in relation to the age- and education-adjusted reference sample using standard values (z-values). Previous reports provide evidence for an improved correct classification of normal and demented patients in the CERAD-Plus compared to CERAD alone [52].

#### 2.3.2. The Modified German version of the Physical Activity Readiness Questionnaire (PAR-Q)

Subjects with existing cardiovascular and pulmonary conditions have increased risk of cardiovascular events during physical exertion [53]. Therefore, the modified German version of the Physical Activity Readiness–Questionnaire (PARQ) was used to screen the physical-activity readiness status of the recruited participants. Participants responded to nine yes/no questions (e.g., if they have previously experienced heart conditions, breathing problems, high blood pressure, pain in the chest, falling down because of dizziness or loss of consciousness, joint problems, cold or fever illnesses, pregnancy) [44]. In the case that at least one of the nine contraindications applied to the participant, a doctor was consulted to decide whether the person was qualified to participate.

#### 2.3.3. Physical Performance and Heart Rate Data

Physical performance and heart rate data were recorded using the Polar Team Pro system (Polar Team Pro, Germany). The Polar Team Pro system (Polar, Kempele, Finland) consists of the Polar Team Pro software that run on a tablet PC, and commercially available wireless Polar HR sensors (Polar H10). The Polar Team Pro system allows us to simultaneously capture data of the internal load (HR (minimum, maximum, average), time in HR zones (zone 1 to 5), beat to beat interval (maximum and average RR interval), the root mean square of successive differences between normal heartbeats (RMSSD)) and external load (covered distance (total and distance/minute), speed (maximum and average)), as well as the training load score of all the participants in each test session. The electrodes of the Polar H10 were moistened with room temperature water prior to being placed on the xiphoid process of the sternum, with the chest strap fitted around the participant’s chest (just below the chest muscles) [54]. The electrocardiography (ECG)-unit of the Polar H10 records electric signals of the heart with a frequency of 1000 Hz and has been previously validated against the 3-lead ECG, which is the gold standard in this field [55]. Thus, the Polar H10 fulfills the technical requirements to quantify HRV, as defined by several methodological guidelines (e.g., measurement frequency > 200 Hz) [56,57,58]. In addition to an ECG unit, the Polar H10 entails a global positioning sensor (GPS), an accelerometer, a gyroscope, and a digital compass recording data at 200 Hz and used to determine the measures of external load [59].

Data collected by the Polar H10’s chest strap sensor were transferred to the associated Polar Team Pro software and then exported to a customized Microsoft Excel spreadsheet. Prior to analysis, the RR intervals were manually corrected for ectopic beats. When identified, ectopic beats were replaced with the average of the two adjacent RR intervals [55]. No specific filters have been used during this process. Participants’ VO_2_ max and maximal HR used for user profiling were determined during a spiroergometric examination, which was accompanied by medical specialists, before the start of the experimentation. We used the following protocol specifications for the spiroergometric assessment: (i) starting workload of 0 Watt, incremental workload of 25 Watt, stage length of 3 min, and cadence of 60–70 revolutions per minute. In the current study, the following termination criteria have been used: (a) no change in HR with an increase in exercise workload/intensity; (b) a Received Perception of Exertion (RPE) of at least 18; (c) a respiratory exchange ratio RER ≥ 1.10; (d) blood lactate concentration ≥ 8.0 mmol/L; (e) or a plateau in oxygen consumption corresponding to an increase of less than 150 mL in oxygen uptake despite an increase in workload. These termination criteria are in accordance with previously established guidelines [60,61].

Detailed descriptions of the physical performance and HR parameters selected from the Polar Team Pro system and analyzed are presented in Table 1.

### 2.4. Statistical Analysis

Data are presented as mean ± standard deviation (SD) and were analyzed using the Statistica software (StatSoft, France; version 10). For variables for which the normality of the distributions was confirmed using the Shapiro–Wilk test, data were analyzed using a one-way analysis of variance (ANOVA) (Time). When appropriate, post hoc pairwise comparisons were performed, and results were interpreted using a Bonferroni correction. For variables for which normality was not confirmed, data were analyzed using a Friedman nonparametric ANOVA and post hoc comparisons were performed using a Wilcoxon test. Effect sizes for the normally distributed variables were calculated as partial eta-squared (ƞp^2^). Partial eta-squared values of 0.01, 0.06, and 0.13 represented small, moderate, and large effect sizes, respectively. For the non-normally distributed variables, effect sizes were estimated by Kendall’s coefficient of concordance. Significance for all analyses was accepted at the level of *p* < 0.05. Exact *p* values have been given; results given as “0.000” in the statistics output have been reported as “<0.0005”.

In order to calculate the gain or decrease between test sessions, difference scores (Δ) from test session 1 to test session 2 (Δ = T2–T1), and from test session 2 to test session 3 (Δ = T3–T2) were calculated for all parameters. Additionally, based on tests and re-tests, the standard error of measurement (SEM) was established for HR- and HRV-related parameters, as well as for physical performance and strenuousness indicators, as previously described [63,64]. Two times the SEM is considered the threshold for a true physiological adaptation beyond the results expected from technical and/or biological variability [65,66]. Therefore, responsiveness to 8 weeks’ training was defined as changes that exceeded two times the SEM in favor of beneficial changes, while responsiveness to 4 weeks’ detraining was defined as changes that exceeded two times the SEM in favor of disadvantageous changes. The responsiveness threshold in each selected parameter as well as the prevalence of responders and non-responders to training and detraining have been reported in the results section.

## 3. Results

### 3.1. Study Population

One hundred and eighteen participants were screened and 28 were deemed eligible to participate in the DIADEM intervention. Thirteen were allocated to the control group, and 15 to the intervention group. One participant in the intervention group dropped out during the training intervention due to a medical problem (back pain). Two other participants missed one testing session and as a result were excluded. As the control group did not receive a Fitness-Dance training program, the Polar team was only used for the intervention group to track their physical performance and HR-related parameters during the training/test sessions. Therefore, in the present study data 12 participants (6 males and 6 females) from the intervention group were included in the final analysis. Based on the effect size of 0.521 previously reported for the effect of 8-week concurrent strength and aerobic Training on HR in Older Adult (Morente-Oria et al. [33], the present sample size (12 participants) can allow a statistical power of 0.95 for an α value of 0.05, as calculated by G * power software (version 3.1.9.2; Kiel University, Kiel, Germany) for an ANOVA.

**Figure 2:**
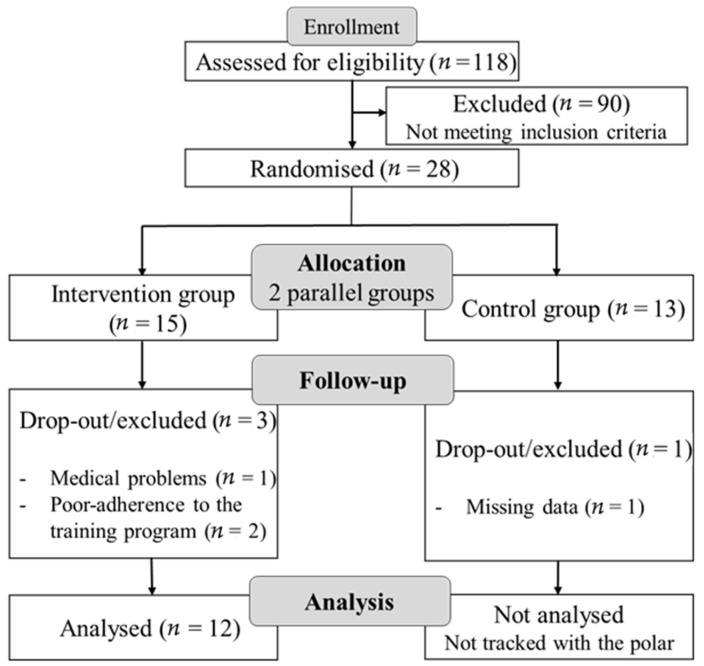
CONSORT flow diagram.

Figure 2 shows the flowchart of a subject’s screening and participation.

Characteristics of the included participants are shown in Table 2.

### 3.2. Effect of Training-Detraining on Min, Max and Avg HR

**Figure 3:**
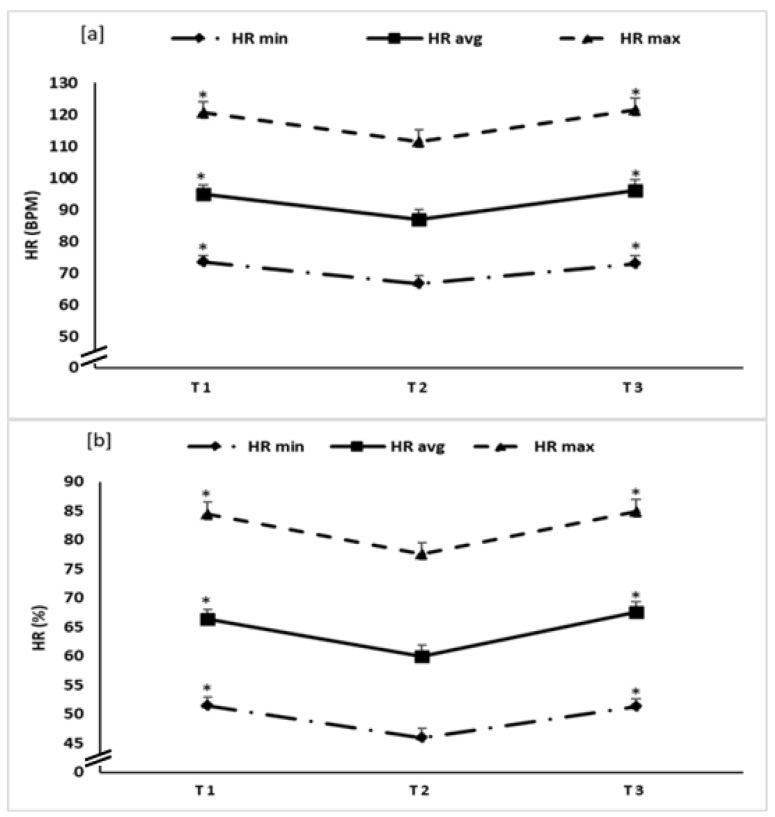
Heart rate (HR) (calculated as bpm: (**a**), and as % of the HRmax: (**b**)) recorded during the first (T1), second (T2) and third (T3) test sessions. *: significant difference compared to T2.

Statistical analysis revealed a significant main effect of time for HR min, HR max and HR average (Figure 3) expressed as bpm or %, with F values ranging between 10.93 to 15.93, *p* < 0.0005 for all parameters, and a np2 range between 0.49 and 0.59.

The post hoc analysis showed that all variables significantly decreased from T1 to T2 with *p* < 0.0005 for HR min (Δ = −7 ± 5 and −6 ± 4, when respectively expressed as bpm and %), *p* = 0.002 and 0.0009 for HR avg respectively expressed as bpm, and % (Δ = −8 ± 8 and −6 ± 6), and *p* = 0.001 for HR max (Δ = −9 ± 8 and −7 ± 6, when respectively expressed as bpm and %).

From T2 to T3, post hoc analysis showed a significant increase in all variables with *p* = <0.0005 for HR min (Δ = 6 ± 5 and 5 ± 4, when respectively expressed as bpm and %), *p* = 0.0009 and <0.0005 for HR avg respectively expressed as bpm, and % (Δ = 9 ± 8 and 8 ± 5), and *p* = 0.0008 for HR max (Δ = 10 ± 8 and 7 ± 5, when respectively expressed as bpm and %).

There was no significant difference between T1 and T3 in any of the tested parameters (*p* > 0.05).

### 3.3. Effect of Training-Detraining on RR Intervals, HRV (RMSSD) and Training Load

**Figure 4:**
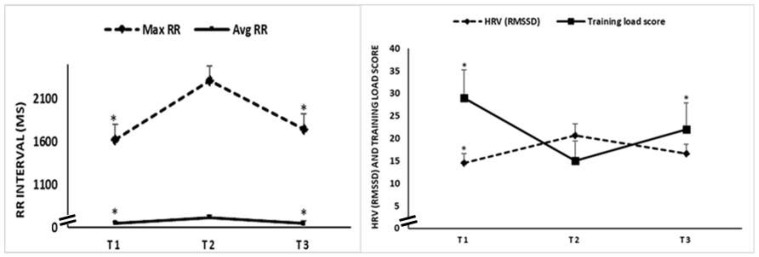
Max RR, Avg RR, HRV (RMSSD) and training load score recorded during the first (T1), second (T2) and third (T3) test sessions. *: significant difference compared to T2.

Statistical analysis revealed a significant main effect of time on Max RR (test = 10.34; *p* = 0.005; Kendall’s W = 0.43), Av RR (F = 6.83; *p* = 0.004; np^2^ = 0.38), HRV (RMSSD) (test = 8.52; *p* = 0.01; Kendall’s W = 0.35), and training load score (test = 6.55; *p* = 0.04; Kendall’s W = 0.27) (Figure 4).

The post hoc analysis revealed that from T1 to T2, all HRV related variables increased, with *p* = 0.006 for Max RR (Δ = 689 ± 545 ms), *p* = 0.01 for Av RR (Δ = 64 ± 76 ms), and *p* = 0.002 for HRV (RMSSD) (Δ = 6.1 ± 4.9 ms), while training load score decreased, with *p* = 0.02 (Δ = −15 ± 19).

From T2 to T3, changes were reversed, with (i) a significant decrease registered for Max RR (*p* = 0.02, Δ = −570 ± 699 ms) and Avg RR (*p* = 0.009, Δ = −68 ± 78 ms), and (ii) a significant increase for training load score (*p* = 0.03, Δ = 7 ± 10). However, the registered decrease for HRV (RMSSD) (Δ = −5 ± 11 ms) was not significant.

There was no significant difference between T1 and T3 in any of the tested parameters (*p* > 0.05).

### 3.4. Effect of Training-Detraining on Percentage of Time in HR Zones

The percentages of time in HR zones are presented in Table 3. Statistical analysis showed that from T1 to T2, % of time in HR zone 1 significantly increased, while % of time in HR zone 3 decreased (*p* = 0.002). From T2 to T3, these changes were reversed, with a significant decrease registered for % of time in HR zone 1 (*p* = 0.006), and a significant increase in % of time in HR zone 3 (*p* = 0.002) and zone 4 (*p* = 0.01).

Time in HR zone 2 and zone 5 did not differ significantly between the three test sessions (*p* > 0.05).

### 3.5. Physical Performance Indicators during the Three Test Sessions

Distance and speed values are presented in Table 4. Statistical analysis showed a significant increase in total distance (*p* = 0.008), distance/min (*p* = 0.02), and average speed (*p* = 0.01) from T1 to T2. From T2 to T3, these changes were reversed, but this decrease was only significant for total distance (*p* = 0.007). Maximum speed did not differ significantly between the three test sessions (*p* > 0.05).

### 3.6. Responsiveness to Training/Detraining Adaptations

The prevalence of responders and non-responders to the 8 week training program and to the 4 week detraining are presented in Table 5. The lower prevalence of non-responders to the beneficial training program was registered for HR min and RMSSD (8%), followed by HR avg and total distance with 17%, and HRmax, max, and avg RR and avg speed with 25%. The higher prevalence was registered for the strenuousness indicator (50%). Regarding the responsiveness to the detraining period, a lower prevalence of responders was registered for HR min and max (83 to 92%), followed by HR avg and avg and max RR with 67–75%, and RMSSD and total distance with 58%. A lower prevalence was registered for the avg speed and strenuousness indicator (42%). Individual responsiveness scores can be found in supplementary file (Figures S1 and S2).

## 4. Discussion

The aims of this study were to investigate whether eight weeks of Fitness-Dance training can improve physical performance and induce cardiac adaptations in older adults with mild cognitive impairment, and if such adaptations can persist following four weeks of COVID-19-induced detraining. Main findings showed that eight weeks of combined Fitness-Dance training induced beneficial cardiac adaptations by decreasing HR (min, avg and max) and increasing HRV-related parameters (max and avg RR intervals and RMSSD) in response to a similar training session. Importantly, participants spent more time in comfortable HR zones and showed lower strenuousness (load scores), despite the higher covered distance and speed. Given that external load and internal load are intertwined [67,68,69], especially considering that a higher external load (higher covered distance and average speed at t2 compared to t1) could be achieved with a lower internal load (lower mean heart rate) to buttress the positive influence of the Fitness-Dance training on physical capacity in older individuals with MCI. However, these positive changes were reversed after four weeks of COVID-19 induced detraining.

In general, the results of HR adaptations following the eight weeks of the combined exercise training program are comparable to previously reported findings in the general population, as well as in older adults. In a recent comprehensive systematic review and meta-analysis of 191 studies assessing HR adaptation following regular physical exercise, Reimers et al. [70] indicated that, in the general population, endurance-based training, as well as combined endurance and strength based-training, significantly decreased the resting HR (2.7 to 5.8 bpm). Specifically, in older adults (>60 years old), previous studies investigating eight-week endurance [71,72,73,74,75] or strength-based [76,77,78] training programs (frequency of 1.5 to 3 session/week), showed a beneficial effect for only endurance-based training programs, with a significant reduction of resting HR ranging from 4.5 to 8 beat/min. Regarding the effect of combined endurance and strength training programs on the resting HR of older adults, previous studies have reported a beneficial effect (reduction of 2.4 to 6.1 beat/min) from 12 to 25 weeks of training (frequency of 2 to 3 session/week) [79,80,81]. However, no data are available on the effect of 8 week combined training programs. The present study confirms the beneficial effect of combining aerobic and strength-based training programs on the cardiac adaptation of older adults with MCI, and demonstrated that eight weeks of Fitness and Dance training (the fitness part entails aerobic and strength exercises) reduced the resting HR by ≈7 beat/minutes, as well as the average and maximum HR measured during the post-test session (T2) (≈−8 and ≈−9 beat/minutes, respectively). Consequently, older adults with MCI performed the post-test session in a more comfortable HR zone (time in HR zone 1 increased by ≈25% vs. time in HR zone 3 decreased by ≈17%), while higher covered distance and speed were registered.

Concerning the HRV-related parameters, previous studies have primarily measured these parameters in a resting state and have revealed contradictory results. Indeed, a significant increase of older adults’ resting HRV has been previously reported following 6 months of aerobic exercise training (31 ± 5 ms) [82], 6 weeks [83] or 6 months [84] of exergaming-based dance training, as well as 12 months of supervised stretching and aerobic exercise training [85]. However, Jakubec et al. [86] and Shen et al. [87] failed to report clear HRV improvements following 6 months or 10 weeks of aerobic exercise training, respectively. Monitoring the HRV-related parameters during exercise sessions, our results show a beneficial effect of shorter training duration (i.e., eight weeks of Fitness-Dance training) as evidenced by the increased values of Max RR, Avg RR and HRV (RMSSD) measured during the post-test session (T2).

Reduced resting-state HRV has previously been shown to be associated with various cardiovascular risk factors (e.g., hypertension, diabetes, and physical inactivity) [88,89,90] and cardiovascular disease (CVD) [91], while greater resting-state HRV has been associated with lower lifetime CVD risk [92]. Similarly, resting HR is known to be inversely related with life expectancy [93] and positively related with cardiovascular and all-cause mortality, as previous reports have reported that a 10 bpm increase in resting HR may increase all-cause mortality by 17% [94]. Stein et al. [95] observed that older adults aged between 65–70 years have the highest decline in cardiac autonomic function, assessed by frequency-domain HRV. Therefore, due to being effective in increasing HRV-related parameters and decreasing resting HR, combined Fitness and Dance training intervention seems to be an efficient training strategy at this specific age to counteract deteriorations in the autonomous nervous system manifested by a decrease in (resting-state) HRV. Thus, a Fitness and Dance training intervention might have the potential to counteract/delay CVD risk and increase life expectancy. Nevertheless, further large studies considering the relation between other lifestyle-associated factors and CVD, such as factors like smoking and alcohol consumption, are necessary to prove this assumption empirically.

Presumably, these beneficial cardiac effects of an eight-week Fitness and Dance training program could be the result of an enhancement of the cardiovascular autonomic control, with possible modification in the sympathovagal balance [41]. Particularly, the lower resting HR and the higher RR intervals and RMSSD following the Fitness-Dance training program may have been caused by an enhanced parasympathetic output. Indeed, Levy et al. [82] showed that at a resting state, increased HRV following six months of aerobic exercise training was accompanied with increased parasympathetic tone in older adults. However, in a more recent study, Bahrainy et al. [96] did not find evidence that a decrease in responsiveness to beta-adrenergic stimulation or an increase in parasympathetic tone contribute to a decrease in resting HR after six months of regular exercise training. Therefore, it can be suggested that training-induced HR decline is most likely due to a decrease in the intrinsic HR, and the exact mechanisms require further investigation.

Following four weeks of COVID-19 induced detraining, our results indicate that the majority of the abovementioned adaptations were reversed, with the values of all HR- and HRV-related parameters during the 3rd test session reaching their baseline levels (1st test session). The present findings are in line with previous studies in young [40,41] and older adults [63]. Indeed, a reversal of cardiovascular autonomic adaptations (e.g., VO2max, HRV and/or parasympathetic activity at the heart level) have been reported following four to eight weeks of detraining in healthy military sailors [40] and sedentary young adults [97]. Similarly, in a recent study investigating older adults with various morbidities (e.g., hypertension, type II diabetes mellitus, Parkinson’s and Alzheimer’s diseases) Coswig et al. [63] showed that the beneficial effects of eight weeks of continuous training (at a moderate-intensity) or interval training (at a moderate or high-intensity) on resting HR were maintained after two weeks of detraining in the interval training groups, and completely reversed after four weeks of detraining in all training groups. The present findings confirm that four weeks of detraining is enough of a period to blind the beneficial effect of an eight-week combined training program (Fitness and Dance) on various HR- and HRV related parameters in older adults with MCI. Taken together, it appears that even with eight weeks on a training protocol, four weeks of detraining generates a loss of training-induced cardioprotective benefits. However, more studies are needed to identify the exact period of retention (e.g., two vs. three weeks) for each training protocol. These unwanted cardiac effects of four weeks’ detraining can be explained by a possible sympatho-vagal imbalance with increased sympathetic or reduced vagal activity. Indeed, based on the paradigm that increased sympathetic tone is associated with decreased parasympathetic tone and vice versa, HRV has been frequently described as mirroring imbalances within the autonomous nerve system [98,99,100]. Nevertheless, given that HRV has also been identified as a surrogate parameter of the complex interaction between the cardiovascular system and brain [101,102], and taking into consideration that the present study investigated older adults with MCI, it is difficult to determine the exact mechanisms underlying the training/detraining-induced HRV adaptation.

Previous reports have emphasized the need to adopt an adequate training protocol to reach better responsiveness [63,65,66], indicating a need for personalized exercise [103]. In this context, Gurd et al. [66] showed a higher rate of young adult non-responders for VO2max, when participants trained for fewer sessions/week (three vs. four session/week). In older adults, Coswig et al. [63] showed a lower overall prevalence of non-responders for a six-min walking test, and body mass following a high intensity (0 and 12%) compared to a moderate intensity interval (53 and 61%) or continuous (39 and 35%) training protocol. To the best of our knowledge, the present study is the first to identify responsiveness for both cardiac and physical performance indicators following training as well as detraining periods in older adults with MCI. Eight weeks of combined Fitness-Dance training resulted in a low prevalence of non-responders for beneficial cardiac and physical adaptations (e.g., 8% for HRmin and RMSSD and 17% for total distance). Contrariwise, a high prevalence of responders was observed after four weeks of detraining for changes in cardiac and physical responses (e.g., 92% for HRmin, and 58% for RMSSD and total distance). All participants exhibited benefits following training and disadvantages following detraining in at least three cardiac parameters, and one parameter of physical performance. These results reinforce the relevance of combining Fitness and Dance training to induce cardioprotective benefits in older adults with MCI and highlight the importance of continuity, because many beneficial adaptations were lost shortly after training cessation.

## 5. Strength and Limitation

The present study is the first to assess cardiac adaptation to combined Fitness and Dance training programs in older adults with MCI by monitoring physical performance indicators and HR responses in a standardized test training session. However, there are several limitations that warrant further discussion. Firstly, the lack of data for the control group could limit the conclusions of the present study, as we cannot rule out the influence of a possible time effect. Secondly, the data of external load (e.g., covered distance and speed) obtained by the Polar Team Pro system should be treated cautiously, as the validity of these measures in an indoor setting has been challenged [59]. Thirdly, several methods have been proposed to analyze interindividual variability, but there is no general consensus on which should be considered the gold standard [104,105]. Thus, our findings concerning interindividual variability should be interpreted in light of this. Fourthly, it is important to include measures of cognitive, balance, and muscle strength tests in future studies, as they are important measures to assess frailty in elderly. In this context, it has been shown that some measures are more resilient against detraining effects (e.g., cognitive performance) than others (e.g., muscular strength) [106,107].

## 6. Conclusions

Eight weeks of combined Fitness and Dance training enhanced various cardiac responses and strenuousness scores during physical effort in older adults with MCI. Therefore, combined Fitness and Dance seems to be an efficient training strategy to promote cardioprotective benefits in this population. Inversely, four weeks of COVID-19-induced detraining reversed these cardiac adaptations. Consequently, to maintain these cardiac benefits, physical training should be continued and a detraining period should be kept to a minimum duration (e.g., two weeks, [63]). In case of strict social-distancing regulations during a pandemic, a home-based intervention may provide an effective way to carry out physical training in older adults to preserve not only their physical but also their mental health [38,108].

## Figures and Tables

**Table 1:** Selected physical performance and heart rate (HR) data from the Polar Team Pro system.

Physical Performance Parameters	
Total distance (m)	Total distance during the training session, particularly during the Dance choreographies and the fitness Dance parts (in meters).
Distance/min (m/min)	Average distance per minute during the training session, particularly during the Dance choreographies and the fitness Dance parts (in meters).
Maximum speed (km/h)	Maximum speed during the training session. Particularly during the Dance choreographies and the fitness Dance parts (kilometre/hour)
Average speed (km/h)	Average speed during the training session, particularly during the Dance choreographies and the fitness Dance parts (kilometre/hour)
Physiological parameters	
Minimum Heart rate (HRmin)	Resting HR before the training session calculated as (bpm) and as % of the HR max (% HR max). A recording duration of 3 min, prior to test sessions, in a standing position was used. In the absence of pathologies or use of pharmaceuticals, a low resting HR indicates, in general, a healthy heart.
Maximal heart rate (HRmax)	Maximal HR during the training session calculated as “bpm” and as % of the HR max (% HR max).
Average heart rate (HRavg)	Average HR during the training session calculated as “bpm” and as % of the HR max (% HR max).
Average RR interval (Avg RR)	Average beat-to-beat interval during the training session (ms). Increase over time means that fitness is improving.
Maximum RR interval (Max RR)	Maximum time between successive heartbeats (beat-to-beat interval) recorded during the training session (milliseconds).
HRV (RMSSD)	The root mean square of successive differences between normal heartbeats (RMSSD) is obtained by first calculating each successive time difference between heartbeats in milliseconds (ms). Each of the values is then squared and the result is averaged before the square root of the total is obtained. The RMSSD reflects the beat-to-beat variance in HR and reflects short-term HR variability HRV [62]. High resting-state HRV is related to improved health and indicates that the heart is functioning well, and that the autonomic nervous system is adapting to the demands placed on it. https://www.polar.com/blog/heart-rate-variability-and-orthostatic-test-lets-talk-polar/ (accessed on 20 April 2021)
Time in HR zone 1	HR zones are a way to monitor the training intensity. There are five HR zones based on the intensity of training with regard to the maximum heart rate. The % of HR max in each zone are as following: Zone 1: 50–60%, Zone 2: 60–70%, Zone 3: 70–80%, Zone 4: 80–90%, Zone 5: 90–100%. (https://www.polar.com/blog/running-heart-rate-zones-basics/). In the present paper, the time in each HR zone was calculated as % of the whole session time (e.g., spending 20 min in zone 1 during a 90 min session → Time in HR zone 1 = 22.22%)
Time in HR zone 2	
Time in HR zone 3	
Time in HR zone 4	
Time in HR zone 5	
Training Load	
Training load score	Training Load includes textual feedback on the strenuousness of a single training session. It is based on the intensity and duration of a training session, with the intensity of a session measured using HR, and the calculation is further affected by personal information such as age, sex, weight, VO2max, and training history. As a participant’s fitness improves, the same training session creates less training load. https://support.polar.com/en/support/the_what_and_how_of_training_load (accessed on 20 April 2021)

**Table 2:** Characteristics of the included participants.

Variable	Mean ± SD	Range
Gender		
Female (%)	50%	
Anthropometric		
Age (years)	73 ± 4.4	67 to 79
Height (m)	1.72 ± 0.08	160 to 183
Body mass (kg)	75.33 ± 6.39	63 to 84
BMI (kg/m2)	25.45 ± 1.97	20.9 to 27.8
CERAD-Plus (z scores)		
Verbal Fluency	−0.38 ± 1.07	−1.51 to 2.44
Boston Naming	−0.40 ± 0.74	−1.59 to 1.04
MMSE	−2.26 ± 0.67	−3.31 to −0.66
Word List Learning	−1.41 ± 0.74	−2.27 to −0.11
Word List Recall	−1.27 ± 0.99	−2.75 to 0.20
Word List Intrusions	−0.46 ± 1.32	−2.46 to 0.86
Word List Savings	−0.97 ± 1.16	−2.73 to 1.01
Word List Recognizing	−0.83 ± 1.11	−2.00 to 0.97
Figures Drawing	−0.23 ± 1.38	−2.63 to 1.02
Figures Recall	−0.67 ± 1.35	−2.35 to 1.17
Figures Saving	−0.40 ± 1.31	−2.18 to 2.01
TMT-A	−0.51 ± 0.84	−2.12 to 0.98
TMT-B	−0.59 ± 0.86	−1.51 to 0.96
S Words	0.24 ± 1.15	−1.49 to 1.67
PAR-Q		
Yes responses	1.67 ± 0.89	0–3
No responses	7.33 ± 0.89	3–9
Cardiorespiratory fitness		
VO_2_max (l/min)	23.08 ± 7.86	14 to 34
HR max (beat/min)	143.27 ± 18.17	118–169

MMSE: Mini–Mental State Examination, TMT-A: Trail Making Test A, TMT-B: Trail Making Test B, VO_2_max: maximal oxygen consumption, HR: Heart Rate.

**Table 3:** Percentages of time in HR zones recorded during the first (T1), second (T2) and third (T3) test sessions.

	T1	T2	T3	Δ from T1 to T2	Δ from T2 to T3	Friedman ANOVA	*p* Value	Effect Size
% time in HR zone 1 (50–59%)	19.4 ± 19.2 *	44.0 ± 29.7	21.1 ± 19.5 *	25 ± 25	−23 ± 25	test = 16.16	<0.0005	0.67
% time in HR zone 2 (60–69%)	47.6 ± 15.0	42.4 ± 19.0	49.8 ± 17.2	−05 ± 24	07 ± 25	test = 1.16	0.55	0.04
% time in HR zone 3 (70–79%)	29.1 ± 21.6 *	12.5 ± 13.6	24.8 ± 20.8*	−17 ± 17	12 ± 13	test = 18.16	<0.0005	0.75
% time in HR zone 4 (80–89%)	03.7 ± 04.0	01.3 ± 01.6	03.7 ± 02.7 *	−02 ± 04	02 ± 02	test = 8.41	0.01	0.35
% time in HR zone 5 (90–100%)	0.1 ± 0.4	00	00	−0.1 ± 0.4	00	test = 2.0	0.36	0.08

*: significant difference compared to T2.

**Table 4:** Distance and speed recorded during the first (T1), second (T2) and third (T3) test sessions.

	T1	T2	T3	Δ from T1 to T2	Δ from T2 to T3	ANOVA	*p* Value	Effect Size
Total distance [m]	712.0 ± 409.5 *	1049.9 ± 493.4	702.6 ± 454.1 *	338 ± 290	−347 ± 410	F = 7.64	0.003	0.41
Distance/min [m/min]	08.4 ± 4.6 *	12.4 ± 06.1	10.2 ± 06.4	04 ± 04	−02 ± 05	F = 4.26	0.02	0.27
Maximum speed [km/h]	07.3 ± 2.7	07.8 ± 02.3	07.1 ± 02.3	0.5 ± 03	−0.7 ± 02	test = 1.16	0.55	0.04
Average speed [km/h]	0.5 ± 0.3 *	0.8 ± 0.4	0.6 ± 0.4	0.3 ± 0.2	−0.2 ± 0.3	F = 5.45	0.01	0.33

*: significant difference compared to T2.

**Table 5:** Prevalence of responders and non-responders to training and detraining adaptations.

Responsiveness to Training/Detraining Adaptations									
Participants Number	HR (bpm)			HRV Related Parameters (ms)			Physical Performance and Strenuousness Indicators		
	HR Min	HR Avg	HR Max	Max RR	Avg RR	HRV (RMSSD)	Total Distance (m)	Avg Speed (km/h)	Load Score
Responsiveness to 8 week Fitness-Dance training									
RT	−1.51	−2.26	−2.01	241.85	22.01	1.3	71.16	0.07	−11.9
Responders	11	10	9	9	9	11	10	9	6
Non-Responders	1	2	3	3	3	1	2	3	6
Non-responders %	8%	17%	25%	25%	25%	8%	17%	25%	50%
Responsiveness to 4 week COVID-19-induced detraining									
RT	1.11	2.01	2.21	−332	−24.9	−2.5	−129	−0.08	1.59
Responders	11	9	10	8	9	7	7	5	5
Non-Responders	1	3	2	4	3	5	5	8	8
% Responders	92%	75%	83%	67%	75%	58%	58%	42%	42%

RT: Responsiveness threshold; NB: Prevalence of non-responders was calculated to evaluate responsiveness to training (beneficial effect), while prevalence of responders was calculated to evaluate responsiveness to detraining (unwanted effect).

## Data Availability

Data are available from the corresponding author upon reasonable request.

## Supplementary Materials

The following are available online at https://www.mdpi.com/article/10.3390/ijerph18115930/s1, Figure S1: Individual responsiveness scores to 8 weeks Dance-training training program, Figure S2: Individual responsiveness scores to 4 weeks Covid-19 induced detraining.
