# Supplementary material for: Four Weeks of Detraining Induced by COVID-19 Reverse Cardiac Improvements from Eight Weeks of Fitness-Dance Training in Older Adults with Mild Cognitive Impairment
Source: Int J Environ Res Public Health. 2021 May 31;18(11):5930. doi: 10.3390/ijerph18115930 (PMC8198940; doi:10.3390/ijerph18115930)
Supplement: Supplementary file 1 [file ijerph-18-05930-s001.zip › ijerph-1213237-supplementary.pdf]

Supplementary Materials:

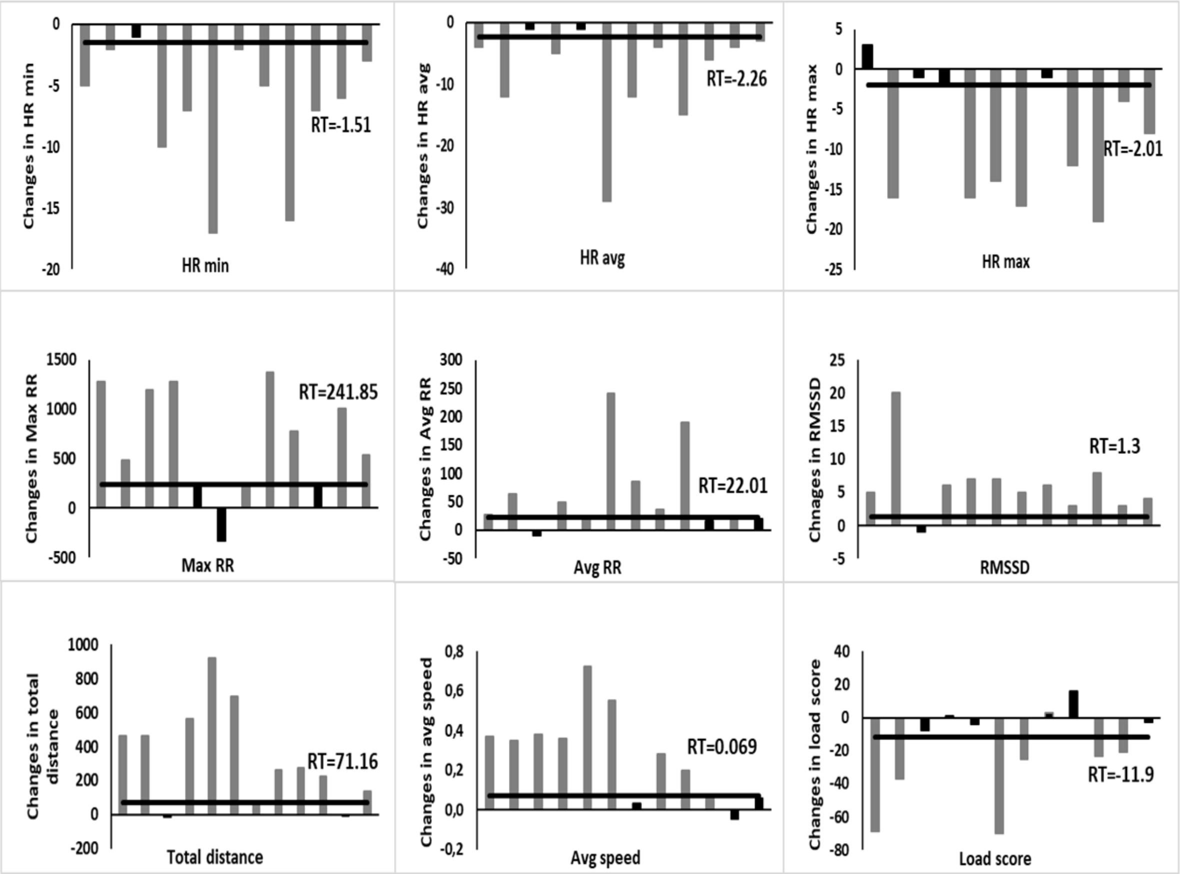

Figure S1. Individual responsiveness scores to 8 weeks Dance-training training program.

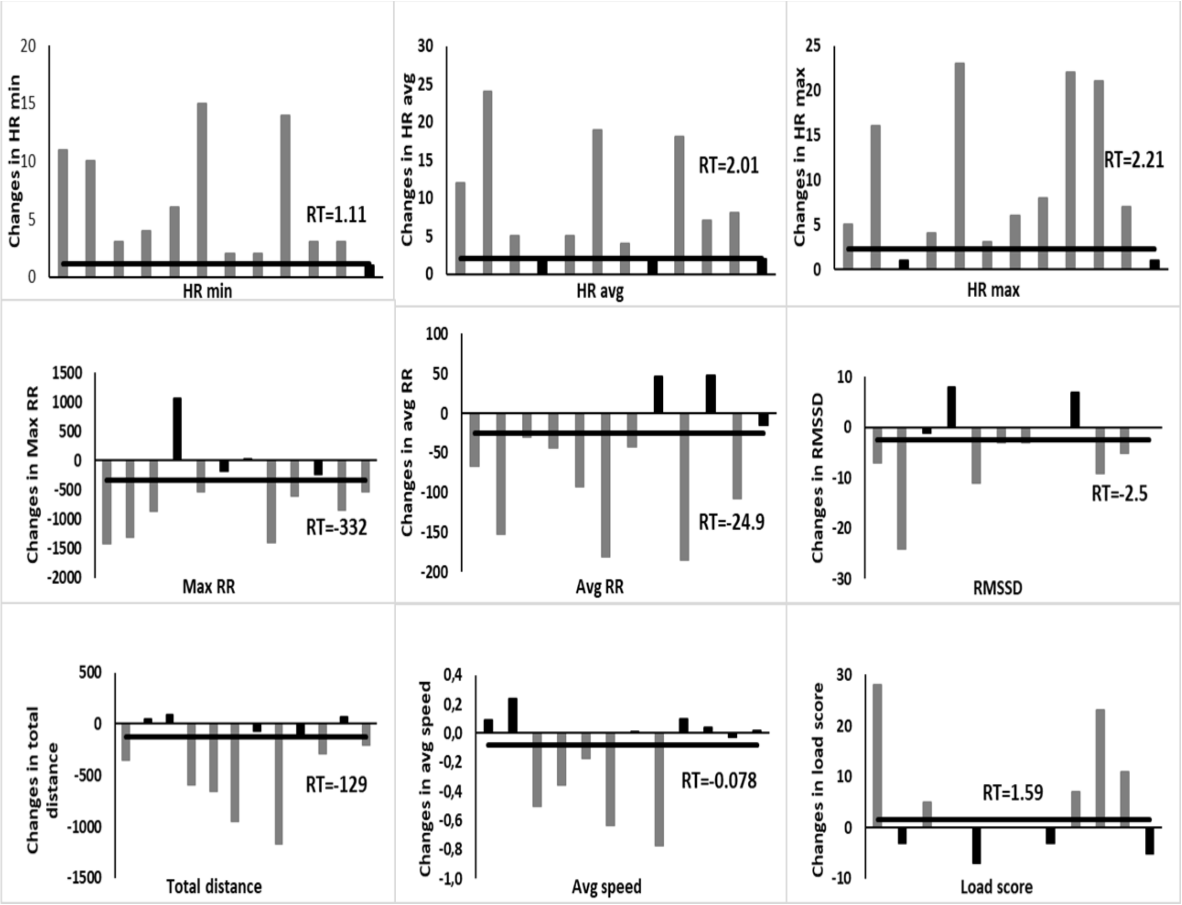

Figure S2. Individual responsiveness scores to 4 weeks Covid-19 induced detraining
